# Alterations in the hippocampus and thalamus in individuals at high risk for psychosis

**DOI:** 10.1038/npjschz.2016.33

**Published:** 2016-09-28

**Authors:** Fabienne Harrisberger, Roman Buechler, Renata Smieskova, Claudia Lenz, Anna Walter, Laura Egloff, Kerstin Bendfeldt, Andor E Simon, Diana Wotruba, Anastasia Theodoridou, Wulf Rössler, Anita Riecher-Rössler, Undine E Lang, Karsten Heekeren, Stefan Borgwardt

**Affiliations:** 1 Department of Psychiatry, University of Basel, Basel, Switzerland; 2 The Zurich Program for Sustainable Development of Mental Health Services, Psychiatric Hospital, University of Zurich, Zurich, Switzerland; 3 Medical Image Analysis Centre, University of Basel, Basel, Switzerland; 4 Specialized Early Psychosis Outpatient Service for Adolescents and Young Adults, Department of Psychiatry, Bruderholz, Switzerland; 5 Department of Psychosis Studies, King’s College London, Institute of Psychiatry Psychology and Neuroscience, London, UK

## Abstract

Reduction in hippocampal volume is a hallmark of schizophrenia and already present in the clinical high-risk state. Nevertheless, other subcortical structures, such as the thalamus, amygdala and pallidum can differentiate schizophrenia patients from controls. We studied the role of hippocampal and subcortical structures in clinical high-risk individuals from two cohorts. High-resolution T_1_-weighted structural MRI brain scans of a total of 91 clinical high-risk individuals and 64 healthy controls were collected in two centers. The bilateral volume of the hippocampus, the thalamus, the caudate, the putamen, the pallidum, the amygdala, and the accumbens were automatically segmented using FSL-FIRST. A linear mixed-effects model and a prospective meta-analysis were applied to assess group-related volumetric differences. We report reduced hippocampal and thalamic volumes in clinical high-risk individuals compared to healthy controls. No volumetric alterations were detected for the caudate, the putamen, the pallidum, the amygdala, or the accumbens. Moreover, we found comparable medium effect sizes for group-related comparison of the thalamus in the two analytical methods. These findings underline the relevance of specific alterations in the hippocampal and subcortical volumes in the high-risk state. Further analyses may allow hippocampal and thalamic volumes to be used as biomarkers to predict psychosis.

## Introduction

Structural brain alterations, as assessed with magnetic resonance imaging (MRI), are commonly reported in schizophrenia patients. The most frequently replicated findings are an increase in ventricle size and a reduction in hippocampal volumes.^1^ Furthermore, meta-analyses of whole brain or region of interest analyses have identified reductions in hippocampal volume in subjects at clinical high risk (CHR) for psychosis already.^2,3^ Volumetric alterations are therefore present before the onset of psychosis and can be studied in CHR individuals with minimal confounding effects of medication and disease progression. The high-risk state is of special interest, as only around 30% of these individuals will eventually develop psychosis^4^ and the identification of these individuals and early intervention might thus prevent or delay transition to full blown psychosis from the CHR state.^5^

The hippocampus and subcortical structures are involved in a variety of tasks, through their interconnection with cortical and other subcortical areas (e.g., learning and memory^6^ and emotional or motivational processing^7^). Aspects of these neuronal brain circuits are at least in part impaired in schizophrenia as well as in the high-risk state already.^8,9^ Moreover, it has been shown that hippocampal and subcortical volumes are moderately to highly heritable in multiplex-multigenerational families affected with schizophrenia.^10^

A worldwide multicentre study with more than 2000 schizophrenia patients and around 2500 healthy controls (HC) assessed hippocampal and subcortical volumes with Freesurfer’s automated segmentation method.^11^ The study showed that the hippocampus, the thalamus, the amygdala and the accumbens were smaller and the pallidum was larger in schizophrenia patients than in HC.^11^ Smaller hippocampal and larger pallidum volumes could be detected by a multi-scanner study in one-tenth of the above population. This study employed automated subcortical segmentation^12^—and automated segmentation of the hippocampus and subcortical volumes is a well-established technique for pooling data from multicentre sites or different scanners.^13,14^ This method allows rapid and robust segmentation with an accuracy, sensitivity and reproducibility comparable to the gold standard of manual segmentation.^15–17^ Although both these studies applied a prospective meta-analysis procedure,^11,12^ the latter also compared the results with a univariate mixed-model regression analysis.^12^ They found that the effect sizes based on the full multisite sample were 13% smaller than those based on the weighted mean effect sizes from each individual site (the prospective meta-analysis).^12^ This result indicates the influence of between-site variance from the use of different MRI scanners.

The present study is a volumetric investigation of all seven subcortical structures (i.e., hippocampus, thalamus, caudate, putamen, pallidum, amygdala, and accumbens) in the CHR state for psychosis acknowledging these methodological facts. We automatically segmented the hippocampus and the subcortical volumes with FSL-FMRIB's Integrated Registration and Segmentation Tool FIRST^18^ in 45 CHR individuals and in 43 HC in a combined cohort from Basel and Zurich. We used linear mixed-model regression analysis to account for scanner effects. As this approach requires similar sample sizes per site for group comparison, the sample sizes were drastically reduced. For comparison, we additionally performed a prospective meta-analysis with 91 CHR individuals and 64 HC. Based on previous meta-analyses,^2,3^ we hypothesized that we would find smaller hippocampal volumes in CHR individuals than in HC.

## Results

### Clinical and demographic characteristics

The subgroup of 88 individuals was matched for gender (*P*=0.20), handedness (*P*=0.99) and site (*P*=0.58). There were significant between-group differences in age (*P*=0.02), education (*P*<0.0001), intelligent quotient (IQ) (*P*=0.04), positive (*P*<0.0001), or negative symptom clusters (*P*<0.0001) and global functioning (GAF;* P*<0.0001; Table 1).

In the larger cohort of 155 individuals no significant differences with respect to gender (*P*=0.14), handedness (*P*=0.68), or IQ (*P*=0.08) were found. There were significant between-group differences in age (*P*=0.03), education (*P*=0.0002), positive (*P*<0.0001), and negative symptom clusters (*P*<0.0001), global functioning (GAF) (*P*<0.0001) and site (*P*<0.0001; Table 2). Among the antipsychotic-naive CHR no significant correlation was detected between any of the significant volumes and psychopathological measures except for a negative trend between the hippocampus and the suspiciousness item (*R*^2^=0.04, *r*=−0.27, *P*=0.09) and a negative trend between the thalamus and the hallucination item (*R*^2^=0.03, *r*=−0.22, *P*=0.096).

### Volumetric differences

With the linear mixed-effects (LME) model to account for site/scanner effects in the subgroup (*n*=88), we detected significant group effects for the volumes of the hippocampus (F=16.91, *P*<0.001, Table 3 and *g*=−0.63, s.e.=0.22, *Z*=−2.90 *P*=0.004, 95% confidence interval (CI)=(−1.06 to −0.21)) and the thalamus (F=10.22, *P*=0.002, Table 3 and *g*=−0.60, s.e.=0.22, *Z*=−2.77, *P*=0.006, 95% CI=(−1.03 to −0.18)). And these effects were also found for the left (F=7.68, *P*=0.0070 and *g*=−0.55, s.e.=0.22, *Z*=−2.51 *P*=0.01, 95% CI=(−0.97 to −0.12)) and right (F=10.35, *P*=0.002 and *g*=−0.56, s.e.=0.22, *Z*=−2.56 *P*=0.01, 95% CI=(−0.98 to −0.13)) hippocampus and the left (F=9.02, *P*=0.004 and *g*=−0.59, s.e.=0.22, *Z*=−2.71 *P*=0.01, 95% CI=(−1.02 to −0.16)) and right (F=10.29, *P*=0.002 and *g*=−0.56, s.e.=0.22, *Z*=−2.56 *P*=0.01, 95% CI=(−0.98 to −0.13)) thalamus separately. High-risk individuals exhibited significantly smaller volumes than HC. These results are corrected for multiple comparisons by employing the conservative Bonferroni-corrected threshold of *P*<0.0071 (two-tailed).

**Figure 1:**
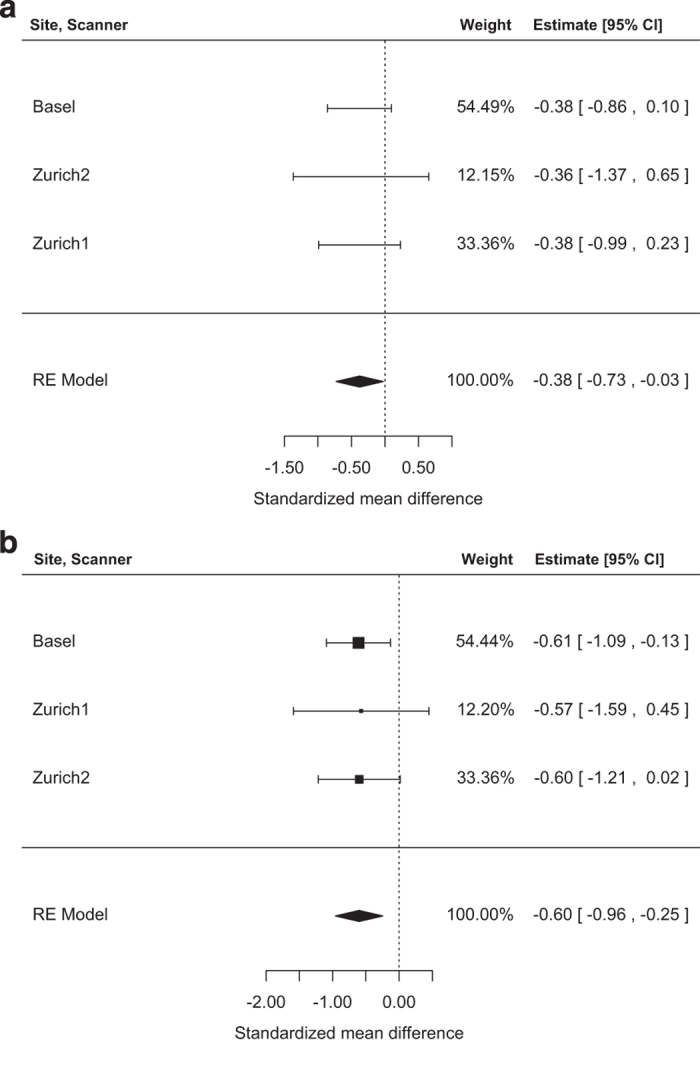
Forest plot of prospective, random effects meta-analyses investigating the difference between: (**a**) hippocampal volumes and group affiliation. (**b**) Thalamic volumes and group affiliation. Negative values represent smaller volumes for CHR than in HC. The dashed line is the zero line of no difference between groups.

The meta-analyses of the hippocampus and the thalamus volumes (n=155) showed smaller volumes for CHR than HC (hippocampus: *g*=−0.38, s.e.=0.18, *Z*=−2.10, *P*=0.04, 95% CI=(−0.73 to −0.03), *Q*(df=2)=0.002, *P*=0.99; thalamus: *g*=−0.60, s.e.=0.18, *Z*=−3.32, *P*=0.001, 95% CI=(−0.96 to −0.25), *Q*(df=2)=0.01, *P*=0.99, Figure 1). Separate effect sizes of group-related comparison for all seven structures (left, right and bilateral volume) and for each site/scanner are presented in Table 4.

## Discussion

In an analysis of automatically segmented hippocampal and subcortical volumes we compared CHR individuals and HC. With the LME model to account for different scanners, we found that the volumes of the hippocampus and thalamus were significantly smaller in antipsychotic-naive CHR individuals than in HC. No between-group differences were observed for volumes of the caudate, putamen, pallidum, amygdala, and accumbens. Extension of PMA to a larger cohort confirmed that hippocampal and thalamic volumes were smaller in CHR than in HC. Moreover, the PMA indicated medium effect sizes for the thalamus and the hippocampus, which were comparable for the thalamus and less for the hippocampus to effect sizes found within the LME approach.

In line with a milestone study of hippocampal and subcortical volumes in schizophrenia patients^11^ and with meta-analyses in CHR populations,^2,3^ our study confirms the findings that hippocampal volumes are smaller in CHR individuals than in HC, although contradictory results exist.^19^ Earlier studies of thalamic volumes showed reductions both in chronic schizophrenia patients and in those with a first episode^20,21^ and especially in antipsychotic-naive schizophrenia patients.^1^ One example of thalamic involvement was shown by automatic pattern classification. Support vector machine analyses exhibited 86% accuracy in classifying CHR from HC and predicted transition to psychosis with 88% accuracy using structural neuroimaging markers only.^22^ The discriminative patterns included hippocampal and subcortical regions, with a prominent role for the thalamus. Thus, specific subcortical changes are present early in psychosis ^1^ or even before the transition to psychosis—in antipsychotic-naive CHR individuals. Moreover, the detected trends for a negative correlation between the hippocampus and suspiciousness (e.g., ref. 23) and the thalamic volumes and hallucination (e.g., ref. 24) should be further investigated as it had been reported in schizophrenia patients. The hippocampus, as one of the most ‘stress-sensitive’ regions of the brain,^25^ and the thalamus, as the main sensory information relay,^26^ might be related to the pathophysiology of schizophrenia.^27,28^ As confirmed by the results of the present study, their structural changes can be detected very early in the CHR population already. Recently, a model for the sudden onset of schizophrenia has been proposed, which attributes a pivotal role to both structures and their interconnection and consolidates the NMDA and dopamine hypotheses.^29^ However, more information is needed to verify this model.

Antipsychotic treatment can attenuate the reduction in the volumes of the subcortical structures,^1^ which is already present in on-going psychosis. In our case, only seven antipsychotic-treated CHR individuals were included in the larger analysis, but this did not reveal any difference from the smaller analysis with only antipsychotic-naive individuals. This small sample size precluded further analysis.

Moreover, in the two studies, 15 and 32 of our CHR individuals were receiving antidepressants at the time of scanning. Antidepressant medication has been previously reported to increase hippocampal volumes in depressive patients.^30^ However, according to the meta-analysis from 15 worldwide centers and almost 9,000 participants, significantly lower hippocampal volumes discriminated patients with major depression from HC irrespective of antidepressant medication.^31^ Thus, we can speculate that the significantly lower hippocampal volumes in CHR could be related to the symptom severity (CHR individuals often suffer from depressive symptoms^32^), independently of antidepressant medication, as is supported by the negative-reported association between hippocampal volumes and negative symptoms in CHR individuals and schizophrenia patients.^33^ However, confounding interactions between clinical characteristics and antidepressant and/or antipsychotic use cannot be ruled out, whereas the subgroup of patients taking medication is likely to be more clinically impaired.

There were other confounding factors we tried to account for, such as the difference in IQ, years of education, and in age. Then, it is known that hippocampal volumes were correlated with educational achievements^34^ and that the maturation of hippocampal and subcortical structures during adolescence and early adulthood is very complex.^35^ Therefore, socioeconomic or other factors might in part mediate the brain morphological changes observed, which are not pertinent for the pathogenesis of psychotic disorders particularly.

Furthermore, owing to slight differences in image acquisition modalities between the two centers, we were forced to pre-process the data for each site separately. This step drastically reduced the sample sizes. To validate our LME results, we performed a prospective meta-analyses of the significantly different volumes, as proposed by the ENIGMA consortium,^11,12,31^ which is an elegant procedure for group-related comparison from different sites. However, we must admit that the generalizability of a meta-analysis with only three samples included is limited. Nevertheless, with the two methods, we obtained the same significant results with medium effect sizes. And as we could only include a small number of CHR with subsequent transition to psychosis (5 in the smaller and 14 in the larger cohort), transition outcome-related brain alterations could not be assessed. Besides, manual segmentation is still considered to be the gold standard, due to its precise delineation of anatomical structures, even though it is costly and time-consuming. Moreover, automated segmentation of the hippocampus and the thalamus with FSL-FIRST was shown to be reliable and correlated well with manual segmentation.^14,16,17,36^ Nonetheless, it has been shown that FSL-FIRST and FreeSurfer generally overestimate large hippocampal volumes and underestimate small volumes compared to manual segmentation.^37^

Furthermore, only one single analysis of genetic covariance between subcortical structural brain phenotypes and risk for schizophrenia has been conducted and this found no correlation.^38^ Nevertheless, future research with larger cohorts should further investigate the possible association between common genetic variants associated with schizophrenia and hippocampal and subcortical brain volumes in CHR populations, as it has been shown that genetic components can influence the volumes of these structures in healthy humans.^39,40^

In summary, in an analysis of 155 individuals, we found smaller hippocampal and thalamic volumes in CHR individuals than in HC individuals. Moreover, we found comparable medium effect sizes for the thalamus and not the hippocampus when assessed by two different analytical methods. These findings demonstrate that these two volumes are already altered in the high-risk state and might incorporate in further analyses as potentially useful biomarkers to predict psychosis.

## Materials and methods

### Participants

For this structural MRI analysis CHR individuals and HC were recruited in two centers: In Basel, as part of the Early Detection of Psychosis research program, FePsy, at the Psychiatry Outpatient Department, University Psychiatric Clinics Basel,^41,42^ and in Zurich, as part of a prospective study on the early recognition of psychosis^43^ within the Zurich Program for Sustainable Development of Mental Health Services (ZInEP), conducted at the Psychiatric University Hospital, University of Zurich.

For details of the recruiting process and clinical assessment as well as inclusion and exclusion criteria, see Smieskova *et al.*^44^/Riecher-Rössler *et al.*^42^ and Theodoridou *et al*.^43^

A total of *N*=91 CHR and *N*=64 HCs from Basel and Zurich were recruited (Table 1). Seven CHR individuals were receiving antipsychotic medication and 32 antidepressants at the time of scanning. In addition, we selected a subgroup of each individual group, in an attempt to have equal numbers of CHR individuals and HC per scanner. This resulted in *N*=45 CHR individuals and *N*=43 HC (Table 2). All individuals of the smaller sample were antipsychotic-naive, whereas 15 of the CHR were receiving antidepressants.

Both studies were approved by the local research ethics committees. All participants provided written informed consent and received compensation for participating.

### MRI acquisition

All anatomical scans from the Basel cohort were performed on a 3T MRI scanner (Siemens Magnetom Verio, Siemens Healthcare, Erlangen, Germany) using a 12-channel phased-array radio frequency head coil. A 3D T_1_-weighted magnetization prepared rapid gradient echo (MPRAGE) sequence was used with the following parameters: an inversion time of 1,000 ms, flip-angle=8 degrees, TR=2 s, TE=3.37 ms, bandwidth=200 Hz/pixel, FOV=256×256 mm^2^, acquisition matrix=256×256×176, resulting in 176 contiguous sagittal slices with 1×1×1 mm^3^ isotropic spatial resolution.

All structural MRI data from Zurich were acquired on a Philips Achieva TX 3-T whole-body MR unit, using an eight-channel head coil. The data were acquired on two identical 3T scanners. A 3D T_1_-weighted fast field echo (FFE) pulse sequence was used to acquire images of the whole brain with the following parameters: TR=8.3 ms, TE=3.8 ms, flip-angle=8 degree, FOV 240×240 mm^2^, voxel size 1×1×1 mm^3^ (reconstructed: 0.94×0.94×1 mm^3^), acquisition matrix=240×240×160, resulting in 160 contiguous slices.

All scans were screened for gross radiological abnormalities by a different neuroradiologist affiliated to each site.

### Image processing

Volumetric segmentation of the hippocampus and the subcortical structures was estimated on T_1_-weighted images using FMRIB's Integrated Registration and Segmentation Tool 5.0.4 (FSL-FIRST).^18^ The different image acquisition modalities (in general, higher image intensities were measured in Zurich) could lead to differences in the segmentation of the volumes. Therefore, we pre-processed the data for each site separately before group comparison. Volumes of all seven structures (accumbens, amygdala, caudate, hippocampus, pallidum, putamen, and, thalamus) were obtained for both hemispheres. To account for non-Gaussian volume distribution, a cube-root transformation was used. The volumes were then normalized with the cube-root of the intracranial volume (ICV) and mean-centered for each site separately, in order to correct for differences in intensities measured in the two sites. After an outlier control (mean±3.5 s.d.), these pre-processed volumetric data were included in the further analyses.

### Statistical analysis

#### Statistical analysis of clinical and sociodemographic data

One-way analysis of variances and *χ*^2^-tests were used to test the distribution between diagnosis group and age, sex, handedness, years of education, IQ, positive symptoms cluster, negative symptoms cluster, each single item of these clusters, GAF, scanner and ICV. Basel and Zurich used different scales for measuring psychotic symptoms. We combined several items of the BPRS with the PANSS outcomes into a positive (suspiciousness (BPRS9, PANSS P6), hallucinations (BPRS10, PANSS P3), unusual thought content (BPRS11, PANSS G9), conceptual disorganisation (BPRS15, PANSS P2)) and a negative (blunted affect (BPRS16, PANSS N1), emotional withdrawal (BPRS17, PANSS N2), motor retardation (BPRS18, PANSS G7)) symptom cluster according to Lyne *et al*.^45^ These statistical analyses were performed with R 3.0.2 software (R Core Team, 2012). Values are presented as mean±s.d. (Table 1). In addition, associations between the bilateral mean volumes (left and right volumes separately corrected for age, gender and years of education by using the *z*-transformed residuals of a linear regression) and clinical symptoms in antipsychotic-naive CHR (positive and negative symptom clusters, all items separately, as well as global functioning) were examined by Pearson correlation analysis.

#### Linear mixed-effects model

The R 3.0.2 software (R Core Team, 2012)^46^ and the packages lme4 (ref. 47) and lmerTest^48^ were used for statistical, group-related analysis. We employed a LME model to assess the relationship between-group affiliation and each volume with left and right volumes combined in one model as separate input. As fixed effects, diagnosis, and site information with interaction terms were entered, as well as age, gender, and education. As random effect, intercepts for subject and hemispheric information were included. Visual inspection of residual plots did not reveal any deviation from homoscedasticity or normality. The significance threshold was set to *P*<0.0071 to correct for multiple comparisons (two-tailed). Moreover, we investigated left- and right-sided volumetric differences using linear regression in R with age, gender, education, and site information as covariate.

#### Prospective meta-analysis

We performed prospective meta-analyses (PMA) of the regions with significant between-group volumetric differences, i.e., hippocampus and thalamus. Data were entered into an electronic database and quantitative meta-analysis was performed using the R 3.0.2 software (R Core Team, 2012). The effect size was calculated using Hedge’s *g*, which provides an unbiased standardized mean difference that incorporates a correction for small sample sizes.^49^ Hedge’s *g* values >0.5 correspond to medium effect sizes. Hedge’s *g* was calculated using data of mean volumes (normalized to ICV and then left and right volumes separately corrected for age, gender, and years of education by using the z-transformed residuals of a linear regression), s.d. and sample sizes. A positive value of the effect size reflected larger volumes for HC than for CHR individuals. We employed a random-effects model with the DerSimonian-Laird estimator, using the metafor package.^50^ Cochran’s Q test was used to evaluate the statistical significance of between-study heterogeneity.

## Figures and Tables

**Table 1:** Demographics and clinical characteristics for the linear mixed-effects model

*Characteristics*	*Clinical high risk (*n*=45)*	*Healthy controls (*n*=43)*	*Statistics*	
Gender M/F (%male)	29/16 (64%)	21/22 (49%)	*χ*^2^=1.59	*P*=0.20
Mean age in years (s.d.)	23.55 (5.28)	26.16 (4.74)	*t*=2.42	*P*=0.02*
Handedness r/l (%left)	41/4 (9%)	39/3 (7%)	*χ*^2^=0.09	*P*=0.99
Years of education (s.d.)	12.27 (2.92)	15.31 (2.91)	*t*=4.71	*P*<0.0001*
IQ (s.d.)	108 (15.58)	115 (14.43)	*t*=2.06	*P*=0.04*
Negative cluster (s.d.)	6.86 (2.86)	3.00 (0)	*t*=−8.97	*P*<0.0001*
Positive cluster (s.d.)	9.07 (3.19)	4.00 (0)	*t*=−10.55	*P*<0.0001*
GAF (s.d.)	58.20 (11.80)	88.17 (4.22)	*t*=15.24	*P*<0.0001*
Scanner ZH1/ZH2/BS	8/11/26	5/14/24	*χ*^2^=1.09	*P*=0.58
Antidepressants no/yes	30/15	43/0	*χ*^2^=15.00	*P*=0.0001*

Abbreviations: F, female; GAF, global functioning; IQ, intelligent quotient; l, left; M, male; r, right; *, significant findings.

Positive symptom cluster=either sum of Suspiciousness, Hallucinations, Unusual Thought Content and Conceptual Disorganisation (BPRS9, BPRS10, BPRS11, BPRS15 in Basel and PANSS P2, PANSS P3, PANSS P6, PANSS G9 in Zurich).

Negative symptom cluster=either sum of Blunted Affect, Emotional Withdrawal and Motor Retardation (BPRS16, BPRS17, BPRS18 in Basel and PANSS N1, PANSS N2, PANSS G7 in Zurich).

**Table 2:** Demographics and clinical characteristics for the prospective meta-analysis

*Characteristics*	*Clinical high risk (*n*=91)*	*Healthy controls (*n*=64)*	*Statistics*	
Gender M/F (%male)	59/32 (64%)	33/31 (52%)	*χ*^2^=2.22	*P*=0.14
Mean age in years (s.d.)	23.70 (5.11)	25.50 (4.76)	*t*=2.24	*P*=0.03*
Handedness r/l (%left)	84/7 (8%)	57/7 (11%)	*χ*^2^=0.17	*P*=0.68
Years of education (s.d.)	12.90 (3.00)	14.89 (2.97)	*t*=3.87	*P*=0.0002*
IQ (s.d.)	108 (15.31)	112 (14.38)	*t*=1.76	*P*=0.08
Negative cluster (s.d.)	6.54 (3.17)	3.00 (0)	*t*=−10.62	*P*<0.0001*
Positive cluster (s.d.)	9.02 (3.52)	4.00 (0)	*t*=−13.53	*P*<0.0001*
GAF (s.d.)	61.05 (14.83)	88.08 (4.15)	*t*=15.19	*P*<0.0001*
Scanner ZH1/ZH2/BS	16/15/60	5/35/24	*χ*^2^=25.25	*P*<0.0001*
Antidepressants no/yes	59/32	64/0	*χ*^2^=26.25	*P*<0.0001*
Antipsychotics no/yes	84/7	64/0	*χ*^2^=3.53	*P*=0.06

Abbreviations: F, female; GAF, global functioning; IQ, intelligent quotient; l, left; M, male; r, right; *, significant findings.

Positive symptom cluster=either sum of Suspiciousness, Hallucinations, Unusual Thought Content and Conceptual Disorganisation (BPRS9, BPRS10, BPRS11, BPRS15 in Basel and PANSS P2, PANSS P3, PANSS P6, PANSS G9 in Zurich).

Negative symptom cluster=either sum of Blunted Affect, Emotional Withdrawal and Motor Retardation (BPRS16, BPRS17, BPRS18 in Basel and PANSS N1, PANSS N2, PANSS G7 in Zurich).

**Table 3:** Results of linear mixed-model analysis

*Variable (nd.f., dd.f)*	*Hippocampus*		*Thalamus*		*Caudate*		*Putamen*		*Pallidum*		*Amygdala*		*Accumbens*	
	*F*	P*-value*	*F*	P*-value*	*F*	P*-value*	*F*	P*-value*	*F*	P*-value*	*F*	P*-value*	*F*	P*-value*
Diagnosis (1, 82)	16.91	<0.001*	10.22	0.002*	4.48	0.04*	3.04	0.09	3.84	0.05	1.67	0.20	6.10	0.02*
Hemisphere (1, 82)	0.01	0.93	0.19	0.67	0.32	0.58	0.36	0.55	0.31	0.58	0.15	0.70	0.85	0.36
Site (2, 82)	0.97	0.38	1.36	0.26	2.33	0.10	0.44	0.65	0.23	0.79	0.60	0.55	0.05	0.95
Diagnosis×hemisphere (1, 82)	0.32	0.57	0.01	0.94	0.0003	0.99	0.04	0.84	0.59	0.44	0.51	0.48	4.95	0.03*
Diagnosis×site (2, 82)	3.09	0.05	0.12	0.89	1.95	0.15	3.09	0.05	0.52	0.59	4.21	0.02*	1.07	0.35
Diagnosis×site×hemisphere (4, 82)	1.19	0.32	0.57	0.69	2.23	0.07	1.70	0.16	1.89	0.12	0.32	0.87	0.84	0.51
Sex (1, 82)	18.93	<0.001*	0.04	0.84	1.78	0.19	0.62	0.43	0.08	0.78	3.42	0.07	0.00	0.98
Age (1, 82)	0.09	0.76	0.01	0.92	10.03	0.002*	1.97	0.16	0.27	0.60	1.79	0.19	2.22	0.14
Education (1, 82)	4.59	0.04*	1.47	0.23	0.02	0.89	0.02	0.88	0.26	0.61	1.13	0.29	0.05	0.82

Abbreviations: dd.f., denominator degrees of freedom; corrected for multiple comparison; nd.f., nominator degrees of freedom; *, significant findings.

**Table 4:** Effect sizes of group-related comparison with bilateral, left and right volume of each stucture

	*Bilateral volume*		*Left volume*		*Right volume*	
	*Hedge's *g	*s.e.*	*Hedge's *g	*s.e.*	*Hedge's *g	*s.e.*
*Hippocampus*						
BS	−0.38	0.24	−0.21	0.24	−0.39	0.24
ZH1	−0.36	0.52	−0.14	0.51	−0.47	0.52
ZH2	−0.38	0.31	−0.52	0.31	−0.13	0.31
						
*Thalamus*						
BS	−0.61	0.25	−0.52	0.24	−0.64	0.25
ZH1	−0.57	0.52	−0.26	0.51	−0.73	0.52
ZH2	−0.60	0.31	−0.69	0.32	−0.45	0.31
						
*Caudate*						
BS	0.17	0.24	0.18	0.24	0.14	0.24
ZH1	−0.40	0.52	−0.26	0.51	−0.50	0.52
ZH2	−0.12	0.31	−0.34	0.31	0.11	0.31
						
*Putamen*						
BS	0.22	0.24	0.18	0.24	0.25	0.24
ZH1	0.17	0.51	0.28	0.51	0.04	0.51
ZH2	−0.38	0.31	−0.58	0.31	−0.14	0.31
						
*Pallidum*						
BS	−0.05	0.24	0.04	0.24	−0.13	0.24
ZH1	0.04	0.51	−0.13	0.51	0.19	0.51
ZH2	−0.16	0.31	−0.07	0.31	−0.20	0.31
						
*Amygdala*						
BS	0.21	0.24	0.17	0.24	0.17	0.24
ZH1	−0.28	0.51	−0.01	0.51	−0.40	0.52
ZH2	−0.35	0.31	−0.30	0.31	−0.29	0.31
						
*Accumbens*						
BS	−0.02	0.24	0.10	0.24	−0.13	0.24
ZH1	−0.56	0.52	−0.29	0.51	−0.58	0.52
ZH2	−0.14	0.31	0.04	0.31	−0.28	0.31

Abbreviations: BS, Basel; ZH, Zürich.

BS and ZH negative effect sizes represent smaller volumes for clinical high-risk individuals than healthy controls. Positive effect sizes represent larger volumes for clinical high-risk individuals than healthy controls.
